# HIV Drug Resistance (HIVDR) in Antiretroviral Therapy-Naïve Patients in Tanzania Not Eligible for WHO Threshold HIVDR Survey Is Dramatically High

**DOI:** 10.1371/journal.pone.0023091

**Published:** 2011-08-19

**Authors:** Christa Kasang, Samuel Kalluvya, Charles Majinge, August Stich, Jochen Bodem, Gilbert Kongola, Graeme B. Jacobs, Mathias Mlewa, Miriam Mildner, Irina Hensel, Anne Horn, Wolfgang Preiser, Gert van Zyl, Hartwig Klinker, Eleni Koutsilieri, Axel Rethwilm, Carsten Scheller, Benedikt Weissbrich

**Affiliations:** 1 Institute of Virology and Immunobiology, University of Würzburg, Würzburg, Germany; 2 Bugando Medical Centre, Mwanza, Tanzania; 3 BUCHS, Mwanza, Tanzania; 4 Medical Mission Institute, Würzburg, Germany; 5 Division of Medical Virology, Department of Pathology, National Health Laboratory Service, University of Stellenbosch, Cape Town, South Africa; 6 Division of Infectious Diseases, Department of Internal Medicine, University of Würzburg, Würzburg, Germany; University of Sao Paulo, Brazil

## Abstract

**Background:**

The World Health Organization (WHO) has recommended guidelines for a HIV drug resistance (HIVDR) survey for resource-limited countries. Eligibility criteria for patients include age below 25 years in order to focus on the prevalence of transmitted HIVDR (tHIVDR) in newly-infected individuals. Most of the participating sites across Africa have so far reported tHIVDR prevalences of below 5%. In this study we investigated whether the rate of HIVDR in patients <25 years is representative for HIVDR in the rest of the therapy-naïve population.

**Methods and Findings:**

HIVDR was determined in 88 sequentially enrolled ART-naïve patients from Mwanza, Tanzania (mean age 35.4 years). Twenty patients were aged <25 years and 68 patients were aged 25–63 years. The frequency of HIVDR in the study population was 14.8% (95%; CI 0.072–0.223) and independent of NVP-resistance induced by prevention of mother-to-child transmission programs. Patients >25 years had a significantly higher HIVDR frequency than younger patients (19.1%; 95% CI 0.095–0.28) versus 0%, P = 0.0344). In 2 out of the 16 patients with HIVDR we found traces of antiretrovirals (ARVs) in plasma.

**Conclusions:**

ART-naïve patients aged over 25 years exhibited significantly higher HIVDR than younger patients. Detection of traces of ARVs in individuals with HIVDR suggests that besides transmission, undisclosed misuse of ARVs may constitute a significant factor in the generation of the observed high HIVDR rate. The current WHO tHIVDR survey that is solely focused on the transmission of HIVDR and that excludes patients over 25 years of age may therefore result in substantial underestimation of the prevalence of HIVDR in the therapy-naïve population. Similar studies should be performed also in other areas to test whether the so far reported optimistic picture of low HIVDR prevalence in young individuals is really representative for the rest of the ART-naïve HIV-infected population.

## Introduction

The World Health Organization (WHO), together with the Joint United Nation Programme on AIDS (UNAIDS) and other partners pursue the goal of providing worldwide access to ART [1]. In most African countries the ART roll-out [2] takes place in a context of a medical infrastructure with limited resources and a low number of available antiretroviral drugs and HIV infection is treated with standardised first- and second-line ART regimens.

Since 2004, a nation-wide care and treatment program has been implemented for HIV-infected individuals in Tanzania. Patients receive their antiretroviral medication free of charge in more than 200 nationwide Care and Treatment Centers (CTCs) and by the end of 2007 more than 165.000 eligible patients have been started on ART. Patients eligible for ART belong to one of the following three groups: a) patients with WHO clinical stage irrespective of their CD4 counts, b) patients with WHO clinical stage 3 and CD4 counts below 350/ml and c) all patients with CD4 counts <200/ml, regardless of their clinical symptoms [3]. The first-line regimens consist of a combination of zidovudine/lamivudine (AZT/3TC) or stavudine/lamivudine (d4T/3TC) together with nevirapine (NVP) or efavirenz (EFV). After therapy failure, a second-line regimen that includes abacavir/didanosine (ABC/ddI) in combination with liponavir or saquinavi boosted with ritonavir (LPV/r or SQV/r) is used [3]. Neither viral load monitoring nor resistance testing are routinely done. HIV-positive pregnant women have been offered participation in a prevention of mother-to-child transmission (PMTCT) programme since 2004 consisting of one single dose of NVP for the mother at onset of labour, followed by a single dose of NVP for the newborn within 72 hours after delivery. Since 2009, a combination therapy of AZT, NVP and 3TC is recommended for PMTCT at sites that have the possibility to offer and monitor this ARV regimen [3].

Although ARV therapy is now available free of charge for eligible patients, ARVs are also sold on the black market in Tanzania. We do not know to which extent this phenomenon contributes to self-medication, especially because these drugs are quite expensive when not being prescribed. However, the fear of stigmatization when receiving ARV regimens at HIV CTCs as well as a general lack of knowledge of HIV treatment guidelines could be factors promoting unadvised medication.

The large-scale availability of first- and second-line ART regimens in a resource-limited area bears the risk of an unnoticed evolution of drug-resistant quasispecies in treated patients and their subsequent transmission into the ART-naïve population [4]. The WHO therefore recommends the monitoring of HIVDR among newly infected individuals in order to estimate the extent to which transmission of drug resistant HIV occurs, classifying HIVDR prevalence into three groups (low: <5%, moderate 5–15%, and high >15%) [5]. Mandatory criteria for inclusion into the WHO-initiated transmitted HIVDR (tHIVDR) surveillance are a) age below 25 years, b) no previous pregnancies, and c) no previous ART use [6]. Their rationale is to exclude people with long-standing infections, infected before ART was widely available in the country, or patients previously exposed to antiretrovirals (ARVs), e.g. through participation in PMTCT programs [5].

So far, the reported prevalence of tHIVDR in eligible patient populations is below 5% across Africa [7], [8], [9], [10], [11], [12] and only for some areas moderate HIVDR rates have been reported in some studies [11]. These rather optimistic findings are in sharp contrast to observations from sub-Saharan Africa, that a significant proportion of HIV patients who start first-line ART encounter early virological failure within the first 12 months of therapy [13], [14], [15]. This may suggest that the true prevalence of HIVDR in patients presenting for ART initiation might be significantly higher than estimated from tHIVDR data.

Therefore, we hypothesized that HIVDR prevalence may differ between people eligible for WHO tHIVDR surveillance and the rest of the HIV-infected population. A systematic underestimation of the prevalence of HIVDR would misguide health care decision makers in resource-limited countries and result in fatal consequences for ART efficacy. In this study we determined the frequency of HIVDR in ART-naïve patients in Tanzania aged below and above the WHO-initiated tHIVDR survey limit of 25 years of age.

## Methods

### Ethics statement

The study was approved by the National Institute for Medical Research (Tanzania), Bugando Centre Ethical Board, and Ministry of Health (Tanzania). All patients gave written informed consent. All clinical investigations have been conducted according to the principles expressed in the Declaration of Helsinki.

### Patients

In this study we investigated samples from HIV patients enrolled in the clinical trial ProCort1 (trial name: “ProCort1”; registry: ClinicalTrials.gov; registration number: NCT01299948). ProCort1 is a randomized, double-blinded placebo-controlled trial testing low-dose (5 mg/day) prednisolone on HIV disease progression in ART-naive patients who are not yet eligible for ART. According to ProCort1 inclusion criteria, patients were HIV positive but ART-naive with CD4+ cell counts >300 cells/µl and no other clinical conditions that require ART. For the ProCort1 trial, a total number of 416 consecutively enrolled HIV patients were initially screened. 90 patients presented as screening failures, mostly (about 80%) because of CD4 counts below 300 cells/µl. The other screening failures were due to TB-coninfections, abnormal blood parameters (HB, ALT, AST, creatinin) or diabetes. Plasma and peripheral blood mononuclear cells (PBMC) were collected from every patient at baseline and at 12 later time points covering a time span of two years. The study was performed at the Bugando Medical Centre in Mwanza (Tanzania).

### Samples and bulk sequencing reaction

75 samples of DNA isolated from frozen PBMC and 55 samples of RNA isolated from frozen plasma collected from 120 randomly-selected patients (from 10 patients both DNA and RNA were isolated) enrolled in the ProCort study were amplified as described [16] by HIV-Pol-specifc PCR or RT-PCR, respectively. A positive PCR reaction was yielded from 46 plasma samples and 48 PBMC samples, corresponding to a recovery rate of 84% (46/55) from plasma and 64% (48/75) from PBMC samples. PBMCs used for these experiments had a low viability, probably a result of suboptimal freezing conditions during transport or storage, which may explain the low PCR recovery rate from these samples. Bulk-sequencing of the PCR products was performed as described [16]. Sequences were submitted to GenBank, accession numbers HM572334–HM572422.

### HIV drug resistance and subtype analysis

For analysis of resistance-associated mutations (RAM), the FASTA files were submitted to Stanfords HIV drug resistance database [17]. The 2009 HIVDR surveillance database [18] was used to identify mutations eligible for the WHO HIVDR surveillance analysis. HIV subtype analysis was performed by phylogenetic analysis of the FASTA files as described [16].

### Therapeutic Drug Monitoring (TDM)

Plasma samples collected at baseline from patients with RAM were subjected to TDM for the detection of Efavirenz, Nevirapine, Nelfinavir, Saquinavir, Atazanavir, and Lopinavir as described [19], [20]. TDM is a well-established investigation in HIV-patients during antiretroviral treatment and part of international guidelines. The methods to determine plasma concentrations of HIV-protease inhibitors (PI) and non-nucleoside reverse transcriptase inhibitors (NNRTI) are highly specific and sensitive. In the HPLC/GC methods used in the study, the limit of detection (LOD) of nevirapine was determined at 2 ng/ml, the lower limit of quantification (LLQ) of nevirapine was reached at a concentration of 10 ng/ml. For efavirenz the LOD was 3 ng/ml, and the LLQ was 25 ng/ml.

### Statistical Analysis

Patients in different age groups were sorted according to the absence or presence of HIVDR in a 2×2 contingency table. Frequency of HIVDR in different age groups was compared using Fisher's exact probability test. P<0.05 was considered as statistically different. 95% confidence intervals (CI) were calculated using GraphPad Prism Software. 95% CIs were truncated at 0.000 if lower CI was negative.

## Results

### Patient characteristics

The sequences analyzed in this study derive from 88 ART-naïve HIV-infected patients in Mwanza, Tanzania, enrolled in the clinical trial ProCort1 (mean age 35.4 years, 78% female) (Table 1). The mean duration since HIV diagnosis was 1.3 years. Twenty patients were younger than 25 years and thus fulfilled the age criteria of the WHO tHIVDR surveillance program, while 68 patients (77%) were aged above 25. This corresponds to the typical age distribution of ART-naïve patients in Mwanza.

**Table 1:** Demographic patient characteristics of the study population.

	PCR-negative	PCR-positive	PCR-positive	PCR-positive
		all	Age<25 years	Age≥25 years
total n = 120	n = 32	n = 88	n = 20	n = 68
**Age (years)**	35.0±12.2	35.2±11.3	22.6±1.5	38.9±10.1
	(21.1–59.0)	(18.6–63.7)	(18.7–24.9)	(25.2–63.7)
**Gender**	77% female	78% female	95% female	75% female
**CD4+ T-Cell count (cells/µl)**	664±299	490±222	534±215	478±223
	(224–1435)	(99–1056)	(140–1037)	(99–1056)
**time since first diagnosis (years)**	1.30±2.75	1.30±1.75	0.77±0.83	1.45±1.92
	(0.05–15.09)	(0.04–9.97)	(0.05–2.99)	(0.04–9.97)
**Sequencing from PBMC (%) compared to plasma**	--	52%	50%	53%
**Clinical stage (CDC) A1**	56%	33%	45%	29%
**Clinical stage (CDC) A2**	38%	51%	45%	53%
**Clinical stage (CDC) B1**	0%	9%	10%	9%
**Clinical stage (CDC) B2**	6%	7%	0%	9%

The study population consisted of 120 ART-naive HIV-1 infected adults. PBMC- or plasma samples from 88 patients yielded amplicons for the bulk sequencing reaction. Data are expressed as means ± S.D. and range in parentheses. Patients with CD4 counts <200/ml at sample date initiated ART if CD4 counts remained below 350/ml four weeks later.

### High frequency of drug-resistant HIV

**Figure 1:**
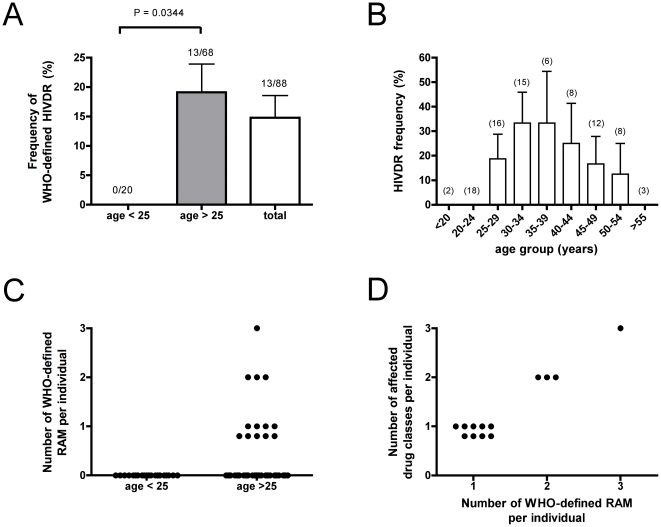
The frequency of HIVDR is age-dependent. HIVDR was determined by bulk sequencing from ART-naïve patients. A.: Frequency of WHO-defined HIVDR in patients aged under 25 years (n = 20, left bar), patients aged over 25 years (n = 68, middle bar), and in the total study population (n = 88, right white bar). B: Frequency of WHO-defined HIVDR peaks in different age groups. Numbers in brackets indicate the number of individuals tested in each age group. Data as means ± S.E.M.. For statistical analysis, Fisher's exact test was performed. Differences with a P<0.05 were regarded as statistically significant. C: Number of WHO-defined RAM per individual (“RAM burden”). D: Number of affected antiretroviral drug classes (NRTI, NNRTI, PI) per individual in relation to the number of WHO-defined RAM per individual.

With bulk sequencing reactions from PBMC-derived DNA (46 cases) and plasma-derived RNA (42 cases) we detected HIV-pol mutations associated with drug resistance in 16 of 88 patients (18.2%; 95% CI 0.100–0.264) (Table 2). Only four of the 16 RAM were initially detected from plasma and 12 from PBMC. In order to investigate if there is any difference in RAM frequency in the two compartments, we sequenced plasma specimens from five patients who were initially tested positive for RAM with PBMC-derived DNA and found the same RAM also in plasma. In five additional cases we retested RAM-negative patients who were initially tested in plasma and found no sequence differences in PBMC (data not shown). Three of the 16 samples had RAM associated with polymorphic positions (T98G; T69N; one patient with both E44D and V118I) that are excluded from WHO tHIVDR surveillance [18]. We hence use the term “WHO-defined HIVDR” to refer to mutations listed by WHO for tHIVDR surveillance [18] and the term “HIVDR” on its own to refer to all RAM according to the Stanford Database, irrespective of their association with polymorphic sites [17]. The frequency of “WHO-defined HIVDR” was 14.8% (13/88, 95% CI 0.072–0.223) in the entire study population (Fig. 1A, white bar).

**Table 2:** HIVDR in the Mwanza cohort.

Age (years)	Sample number	NRT RAM	NNRTI RAM	PI RAM	Affected drug (low-level resistance)	Affected drug (high-level resistance)	TDM	time since HIV test (years)
20.5	TZ.09.032647		A98G^1^		NVP		neg.	0.31
25.4	TZ.08.029746			**D30N**	ATV	NFV	neg.	0.58
26.4	TZ.08.017184	**M41L**			D4T, AZT, ddI, ABC		neg.	1.05
28.0	TZ.08.014495	**M184I**			ABC	3TC, FTC	**EFV**	0.33
30.2	TZ.08.022112	**M184I**	**G190E**		ABC	3TC, FTC, NVP, EFV	neg.	8.3
32.2	TZ.09.001336			**G73S**	SQV, NFV, ATV		neg.	0.28
32.4	TZ.09.001332	**M184I**	**K103N**		ABC	3TC, NVP, EFV, DLV	neg.	0.18
33.2	TZ.08.017176	**M41L**			D4T, AZT, ddI, ABC		**NVP**	2.50
33.7	TZ.08.023280	**V75A**			D4T, ddI		neg.	1.63
36.7	TZ.08.017197	**T215I**	**K103N**	**V82T**	D4T, AZT, ddI, ABC, SQV/r, LPV/r, ATV, NFV	NVP, EFV, DLV	n.d.	2.81
38.7	TZ.09.001323		**G190E**	**G73S**	SQV/r, NFV,ATV, DLV	NVP, EFV	neg.	1.06
42.6	TZ.09.006017			**V82A**	LPV/r, SQV/r, ATV, NFV		neg.	4.60
44.9	TZ.10.002035	T69N^2^			ddI		negative	1.14
46.7	TZ.08.017195		**K103N**			NVP, EFV, DLV	n.d.	0.38
46.8	TZ.10.003309	E44D^3^V118I^3^			3TC		neg.	0.15
51.4	TZ.08.017193		**Y188H**		NVP, EFV, DLV		neg.	0.04

RAM according to the Stanford HIV Drug Resistance Database [17] in 16/88 baseline samples. Mutations associated with a score of 60 were attributed as high-level resistance-associated mutations (RAM) and mutations with a score of 10–35 were attributed as low-level RAM. Mutations listed for WHO HIVDR surveillance [18] are indicated in bold. Superscripted numbers identify the reasons for excluding the respected mutations from the HIVDR list: 1) nonpolymorphic, but at highly polymorphic position; 2) polymorphic position in subtypes B, F, CRF01_AE; 3) polymorphic in multiple subtypes. ABC: Abacavir; ATV: Atazanavir; AZT: Zidovudine; ddI: Didanosine; DLV: Delaviridine; D4T: Stavudine; EFV: Efavirenz; FTC: Emtricitabine; LPV: Lopinavir; NFV: Nelfinavir; NVP: Nevirapine; SQV: Saquinavir; 3TC:Lamivudine [29]. Underlined drugs are part of the local first line regimens, double line underlined drugs are part of local second line regimens. Data derived from 88 sequenced samples. Therapeutic drug monitoring (TDM) was performed from plasma samples collected at baseline for NNRTIs (Efavirenz and Nevirapine) and PIs (Nelfinavir, Saquinavir, Atazanavir and Lopinavir).

### Age-dependent frequency of drug-resistant HIV

The occurrence of RAM was highly correlated with the age (Fig. 1). WHO-defined HIVDR frequency was 0% (0/20) in patients aged under 25 but 19.1% (13/68; 95% CI 0.095–0.28) in patients older than 25 years (Fig. 1A), which is statistically significantly different (p = 0.0344, Fishers exact test).

As depicted in figure 1B, frequency of HIVDR seems to peak in middle-aged patients, although the number of patients in each age-group is too small in order to reach statistical significance. Some of the patients presented with two or three different RAM (Fig. 1C). This “RAM burden”, i.e. the number of different RAM contributing to resistance against ARVs per individual, correlates with impairment of a greater spectrum of classes of ARVs (Fig. 1D).

### Clinical impact of the RAM

**Figure 2:**
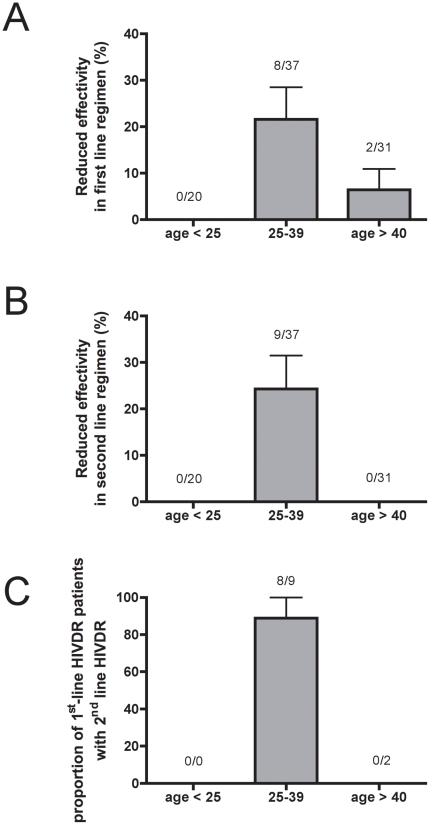
HIVDR affects efficacy of local first- and second-line ART regimens. Effects of HIVDR (high- and low-level resistances) on drugs included in the local antiretroviral regimens. A: Effects on local first-line ART regimen. HIVDR that affects at least one of the drugs included in first-line therapy (AZT/D4T plus 3TC plus NVP/EFV) scored positive. B: Effects on local second-line ART regimen. HIVDR that affects at least one of the drugs included in second-line therapy (ddI/ABC plus LPV/SQV plus RTV) scored positive. C: Proportion of patients with HIVDR that affects first-line ART regimen who also carry HIVDR that affects second-line ART regimen.

The resistance pattern detected could severely limit the options for ART once the patients become eligible for antiviral therapy. About every fifth patient (21.6%; 8/37) in the group 25–39 years has a predicted reduced susceptibility towards the local first-line ART regimens according to Stanford Database (Fig. 2A). Without prior resistance testing, prescribing this regimen would result in insufficient suppression of viral replication, likely leading to a rapid development of further mutations conferring resistance to other components of the regimen and – finally – to complete virological failure. Even more concerning are the findings that the efficacy of the local second-line regimens is also affected by the detected mutation profile: a) 24% (9/37) of the patients aged between 25–39 have RAM that correspond to low- and high-level resistance towards ARVs included in the local second-line regimens according to Stanford Database (Fig. 2B) and b) 8 out of 9 patients (89%) in this age group with predicted reduction of first-line efficacy also have a predicted reduction of second-line efficacy (Fig. 2C). Related to the whole study population, these numbers could translate into complete ART failure (first- and second-line) in about 9% (8/88) of all patients.

### Long-term persistence of RAM

In order to determine the long-term persistence of RAM in the 16 patients with baseline RAM, plasma specimens collected up to 21 month later were analyzed. Three different scenarios were observed: In five patients the initially-detected RAM persisted, in six patients RAM detected at baseline became undetectable later, and in five patients RAM that were undetected at baseline were found in follow-up samples (Table 3).

**Table 3:** Long-term persistence of RAM.

	Sample number	RAM at baseline	Time to Follow-up 1 (months)	RAM at Follow-up 1	Time to Follow-up 2 (months)	RAM at Follow-up 2
	TZ.09.032647	**A98G**	**5**	**A98G**	n.a.	n.d.
	TZ.08.017184	**M41L**	**11**	**M41L**	**18**	**M41L**
persistent RAM	TZ.08.017176	**M41L**	**12**	**M41L**	**21**	**M41L**
	TZ.08.017195	**K103N**	**13**	**K103NS**, Y188HY	**18**	**K103N**
	TZ.10.003309	**E44D, V118I**	**21**	**E44D, V118I**	n.a.	n.d.
	TZ.08.029746	**D30N**	12	none	n.a.	n.d.
	TZ.09.006017	**V82A**	18	none	n.a.	n.d.
RAM disappeared	TZ.08.022112	**M184I, G190E**	15	Q151QR	18	none
	TZ.09.001336	**G73S**	18	V179T	n.a.	n.d.
	TZ.08.023280	**V75A**	12	T69ST	14	T69ST
	TZ.08.017193	**Y188H**	6	L10V	n.a.	n.d.
	TZ.08.022112	M184I, G190E	**15**	**Q151QR**	18	none
	TZ.09.001336	G73S	**18**	**V179T**	n.a.	n.d.
new RAM	TZ.08.023280	V75A	**12**	**T69ST**	**14**	**T69ST**
	TZ.08.017193	Y188H	**6**	**L10V**	n.a.	n.d.
	TZ.08.017195	K103N	**13**	K103NS, **Y188HY**	18	K103N
	TZ.08.014495	M184I	6	no PCR product	n.a.	n.d.
	TZ.09.001323	G190E, G73S	18	no PCR product	n.a.	n.d.
unclear	TZ.09.001332	M184I, K103N	n.a.	n.d.	n.a.	n.d.
	TZ.08.017197	T215I, K103N, V82T	n.a.	n.d.	n.a.	n.d.
	TZ.10.002035	T69N	n.a.	n.d.	n.a.	n.d.

Of the 16 patients who presented with RAM at baseline, we analyzed plasma specimens collected at later time points (“follow-up 1”, “follow-up 2”) for the presence of RAM. We detected three different scenarios, including persistent RAM, disappearing RAM, and newly emerging RAM (some samples appear in more than one scenario). Time to follow-up sample is indicated in months. In some cases, no PCR product from plasma samples could be generated. The investigation for long-term stability of RAM is insofar incomplete as plasma specimens collected at later time points were not available (“n.a.”) for all patients, referred to as “n.d.” (not determined).

### Therapeutic drug monitoring

Baseline plasma samples of 14 patients with RAM were screened for the presence of antiretroviral substances. We detected traces of efavirenz (160 ng/ml, sample number TZ.08.014495) and nevirapine (202 ng/ml, sample number TZ.08.017176) in two samples (Table 2). These drugs are, however, unrelated to the RAM detected in these patients (M184I and M41L). For one patient (TZ.08.017195) in which we detected K103 that confers resistance to EFV or NVP and was stable for at least 18 months, we screened additional plasma samples donated at 3, 6, 12 and 18 months after initial sampling and found no traces of either drug.

### HIV-1 subtype distribution

**Figure 3:**
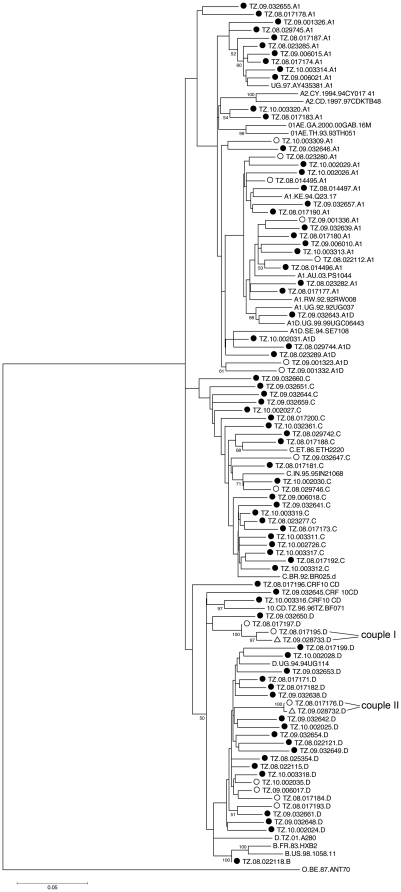
Phylogenetic analysis of RT and PR sequences from the Tanzanian cohort. A neighbour-joining phylogenetic tree [30] was constructed from the 88 patient derived HIV-1 sequences from the Tanzania cohort and the two partner-derived sequences. Reference sequences were obtained from the Los Alamos HIV sequence database. The analyzed 1302 bp region includes the complete Protease and Reverse Transcriptase coding region. The tree was constructed using Mega software version 4, and the evolutionary distances were calculated using the Kimura 2-parameter method. The bootstrap consensus tree was inferred from 50000 replicates and values greater than 70% are indicated on the branch lengths. The scale at the bottom left indicates the calculated genetic distances between the branches of the phylogenetic tree. Circles represent the 88 samples from our cohort. Black-dotted circles are without RAM, open-circled sequences are with RAM, open triangles are sequences with RAM from HIV-infected partners of two study subjects, which were not included in the determination of HIVDR as these patients received ART. Sequences without symbols are subtype reference sequences derived from Los Alamos database. The subtype is indicated at the end of each sequence name. Relative subtype frequency: A1: 34%, A1D: 7%, C: 26%, CRF10_CD: 4%, D: 28%, B: 1%. Sequences isolated from two couples (couple I, couple II) with NVP resistances.

Subtype analysis identified seven different clusters that can be divided into different HIV subtype groups, including A1 (34% of the samples), B (1%), C (26%), D (28%), CRF10_CD (4%) and recombinants A1D (7%) (Fig. 3). These findings are in line with previous reports from the area [10], [21].

## Discussion

The frequency of RAM in our ART-naïve study group is much higher than expected from hitherto reported prevalence rates of below 5% from WHO tHIVDR threshold surveys across Africa [22]. This this difference is probably mainly attributed to the different criteria for choosing patients for HIVDR surveillance: The WHO surveillance criteria define eligible patients as presumably recently infected patients below 25 years of age with CD4+ T-cell counts above 500 cells/µl, and – if female - restricted to women without prior history of pregnancies to avoid PMTCT-induced resistance. Our study population, in contrast, consists of HIV patients with a probably slightly longer history of infection (1.30±1.75 years since diagnosis) with a mean age of 35.4 years, a mean baseline CD4+ T-cell count of 483 cells/µl and amongst females predominantly multigravidae (Table 1).

By applying the age criteria of WHO HIVDR surveillance to our analysis, we would have concluded that the prevalence of drug resistant HIV in the Mwanza region is below 5% similar to what was reported for the Dar es Salaam region in Tanzania in 2008 [23] and from most other HIVDR surveillance sites across Africa so far [7], [8], [9], [10], [11], [12]. However, the true frequency of HIVDR in our study population is much higher: 14.7%, if mutations at polymorphic sites are excluded and even 18.2% if they are included.

This alarmingly high frequency of HIVDR could have been generated by two different ways: a) by direct transmission of drug resistant viruses from sexual partners carrying the resistant virus or b) by selection from the natural pool of quasispecies in each individual patient following undisclosed antiretroviral drug experience. It is reasonable to suggest that both factors may have contributed to the observed high frequency of RAM in our Tanzanian study group.

Intra-patient generated drug resistance in ART-naive patients can either result from episodes of NVP monotherapy of pregnant women during PMTCT or from undisclosed (self-) medication with ARVs. To exclude PMTCT-generated NVP resistances from tHIVDR surveillance, the WHO criteria exclude multigravidae, which results in excluding many, if not the majority of women.

In our study group we identified seven patients with NVP resistance (Table 4). We can exclude PMTCT as a possible cause for the occurrence of the NVP resistance mutation in five of them (71%), because the patients are either male (two cases) or the pregnancies date before HIV infection was diagnosed (three cases). In one additional case we were unable to ascertain the patient's PMTCT status and in only one case we positively identified a patient who actually received PMTCT. Therefore, the vast majority of NVP resistance in our study group is obviously unrelated to PMTCT. The two NVP resistance mutations that can be potentially attributed to PMTCT have, however, no influence on the overall HIVDR frequency in our study group, as the respective patients (TZ.09.001332, TZ.09.001323) present with multiple RAM against other ARVs that are completely unrelated to the Tanzanian PMTCT program. Since these other mutations (M184I and G73S, respectively) must have been acquired independently of PMTCT, there is no reason to exclude these two patients from the analysis.

**Table 4:** NVP resistance and PMTCT.

Sample number	Gender	PMTCT with NVP	Birth dates of children	First HIV diagnosis	NVP resistance caused by PMTCT
TZ.09.032647	female	none	2007	24.08.08	**no**
TZ.08.022112	male	n.a.	n.a.	24.09.04	**no**
TZ.09.001332	female	unknown	unknown	23.08.07	**unclear**
TZ.08.017197	female	none	1989, 1993, 1995	16.04.07	**no**
TZ.09.001323	female	yes	1990, 1992, 1994, 1996, 2004, 2006	26.01.06	**possible**
TZ.08.017195	female	none	1992, 1993, 1995, 1997, 1999	22.05.07	**no**
TZ.08.017193	male	n.a.	n.a.	15.09.05	**no**

Patients with NVP resistance mutations were analyzed regarding PMTCT (with NVP monotherapy) as a possible cause for the emergence of the mutation. PMTCT as a cause for the mutation's appearance is discussed as “possible” if the mother received PMTCT; it is discussed as “unclear” if the PMTCT status and the dates of birth of the children are unknown; for plausibility reasons PMTCT was excluded (“no”) as a trigger of the NVP mutation if the patient is either male or if a female patient presented with unknown PMTCT history combined with the HIV infection being diagnosed only since the date of birth of the youngest child.

As for undisclosed self-medication, all patients who presented with RAM repeatedly reported that they were treatment-naïve (except for those who participated in PMTCT programs) and also their medical records – if available - were negative in this respect, suggesting that not only the majority of NVP mutations but also a great proportion of the other detected mutations were acquired by transmission.

In an attempt to challenge the given disclosure about the patients' drug status, we used TDM as a forensic method in order to determine whether the patient has taken NNRTIs or PIs within the past 1–2 weeks, the time window in which we can detect these drugs in plasma samples [24], [25]. Of the 16 patients who presented with RAM, traces of ARVs were detected in two (Table 2), indicating that at least some of the patients gave a false statement regarding their drug status. We can almost exclude that the patients studied here received ARVs by CTCs, as their health status was still good enough to be eligible for ART and any clinically-advised antiretroviral medication would have been documented in their medical records – which were negative in this regard. To our knowledge, this is the first reported evidence for undisclosed self-medication with ARVs in Africa, a phenomenon that has unfortunately not yet been addressed by systematic analyses. The detected ARVs were, however, not related to the RAM found in these patients, so that the undisclosed medication with ARVs might just have been an epiphenomenon and the detected RAM may nonetheless have been acquired by transmission.

According to WHO criteria, these two patients are not eligible for tHIVDR survey, because they were (as found by experimental evidence) not ARV naïve. Even if we excluded these patients from our analysis, HIVDR frequency would still be 12.5% (11/88) and therefore much higher than anticipated from previous reports from Tanzania [23]. However, we did not exclude the patients from our analysis for the following reasons: a) First and foremost, the aim of our study is not to fulfil current WHO criteria as good as possible and to see how close we can get to previous-published results, but rather to challenge these very criteria by looking into a therapy-naïve population as it is, i.e. not being filtered by additional criteria concerning age or drug naivety (not to be mistaken with therapy naivety!). In our case, the frequency of HIVDR in patients who have never before received medically-advised antiretroviral therapy is in the range of 15% and this constitutes a severe medical problem for future antiretroviral therapy with predefined regimens - no matter how many patients we exclude from our analysis for not fulfilling WHO criteria for whatever reasons. b) The availability of ARVs on the black market is a known phenomenon in many sub-Saharan countries and there is no reason to believe that undisclosed self-medication is a phenomenon restricted to our study population. It is rather plausible to assume that self-medication with ARVs (be it from black-market supplies or by drug-sharing within a family) is inevitably connected to ART roll-out programs and surveys to monitor the prevalence of HIVDR in therapy-naïve patients should not exclude this source of drug resistance. c) WHO criteria do not include forensic methods to control whether the studied populations are really drug-naïve, and to our knowledge, drug monitoring has not been done in any of the tHIVDR surveys published so far.

In five out of the 16 RAM carriers we observed the emergence of new RAM at later time points (Table 3). It seems very unlikely that these newly-detected RAM originate from superinfections with drug-resistant viruses but rather that the emergence of these RAM was either caused by recent misuse of ARVs or - probably the more plausible interpretation – simply reflects a false-negative result of the bulk sequencing method at baseline determination (for discussion of bulk sequencing sensitivity see also below). In three cases the RAM detected at baseline remained stable for more than 18 months. Two of these patients carried the mutation M41L and one carried the mutation K103N. For both mutations a long-term stability has been reported even in the absence of antiviral medication [26].

In four of the 16 patients presenting with RAM, plasma samples could be obtained from their HIV-infected partners. Two of these could be sequenced and had the same RAM as their partners (K103N detected in TZ08.017195 and the partner's sequence TZ09.028733, and M41L detected in TZ08.017176 and the partner's sequence TZ09.028732). In a phylogenetic analysis these sequences clustered in direct neighbourhood to the partner's sequences (marked with “couple I” and “couple II” in Fig. 3). As it is rather unlikely that a potential misuse of an ARV generates exactly the same RAM profile in both partners (many different RAMs can become selected by a given ARV), it is reasonable to assume that the respective RAM was received by transmission, although we cannot say in which direction the transmission occurred.

Irrespective of whether the observed high frequency of RAM is predominantly caused by transmission or by selection with undisclosed medication with ARVs, such a high frequency of RAM in patients who are likely to start ART in the near future translates into an expected reduction of first- and second-line ART regimen efficacy. The full extent of the problem may be even greater, as we generated our data from bulk sequencing reactions, which are known to underestimate the number of RAM, because they fail to reliably detect mutations if the respective quasispecies contribute less than 20–30% to the total viral population [27]. More sensitive techniques, such as real time PCR-based resistance testing, show that bulk sequencing considerably underestimates the true frequency of HIVDR [28].

If the observed level of HIVDR in our study group is representative of other regions of Africa, this would affect the settings in which ART roll-out programmes are being implemented. The current WHO surveillance criteria bear the danger of answering a merely academic question (i.e. the transmission rate of resistant viruses in people aged under 25), whereas the clinical reality has to deal with pre-existing HIVDR in all patients eligible for ART, irrespective of their origin (transmitted or intrapatient-selected) and irrespective of the patient's age. Beyond that, it is questionable whether the under-25 year olds are a representative “sentinel group” for HIVDR transmission in the rest of the population, because important social parameters that presumably affect the rate of resistance transmission, such as promiscuity or the ART status of the sexual partners are likely to significantly differ between different age groups, which may compromise the current resistance surveys. Moreover, the (so far excluded) patients over 25 years of age represent a significant proportion in the group of patients eligible for ART. Over the years at Bugando Medical Centre, the proportion of those older than 25 years amongst patients eligible for ART has been between 70 to 80 percent. This is similar to the age distribution in our study group, which in this respect quite accurately pictures the characteristics of patients seeking for ART at Bugando Medical Center. Our study population was, however, not intentionally designed to represent the whole HIV-infected population in Mwanza, so that an extrapolation of our data to the whole population in Mwanza must be taken with caution. Moreover, the patients investigated in this study have been reenrolled for an interventional clinical trial (“ProCort1”), which may have caused a selection bias (like, for instance, patients who agree to join an interventional trial may or may not be more prone for self-medication with ARVs in the past or to keep information about ARV misuse undisclosed). We therefore refer to the term “frequency” rather than “prevalence” when describing the rate of RAM in our patient population.

WHO recommends the analysis of at least 34–47 specimens (34 if no mutations were found, ≥47 if one or more mutations are detected within the first 34 sequences) of consecutively enrolled patients for the threshold survey method in order to categorize HIVDR as <5%, 5–15% or >15% [18]. Here we analyzed 88 samples and the determined frequency of HIVDR is therefore quite robust. Although we did find a frequency of HIVDR in the range of 5–15% in our study group – a finding that in the context of an expected low HIVDR rate is remarkable in itself – the most important aspect of our study is that this relatively high frequency of HIVDR would have remained completely unnoticed, if we strictly applied the current age criteria (<25) of the WHO threshold survey method to our analysis. The difference in HIVDR between the two age groups was so pronounced that it reached statistical significance despite the relatively low number of only 20 studied patients aged below 25 years. On the other hand, the estimation of a 0% HIVDR frequency in patients <25 years remains relatively uncertain, as the number of analyzed sequences in this subgroup is smaller than recommended.

The most substantial argument in favor of an age restriction of HIVDR surveys to patients younger than 25 years is probably the intention to exclude as much as possible older infections that reflect HIVDR transmission rates of the past rather than the current situation. In our study, the mean time since HIV diagnosis of the patients under 25 years old was 0.77 (±0.19 SD) years with a range of 0.05–2.99 years, compared to 1.45 (±0.23 SD) years for patients aged above 25 years (p = 0.1285, non-significant) (Table 1). Moreover, 10 of 13 (77%) patients with HIVDR had a known infection time within the range of the young age group, and 6 of 13 (46%) had infection times even below the average of the young group (Table 2). This is in fact not very astonishing, as young people in Tanzania are considered to be at substantial risk of HIV infection by sexual intercourse already at the age of 15 [3] which is still ten years away from the 25 years limit. Combined with an asymptomatic phase of infection that can last on the one hand for several years but on the other hand turns into a symptomatic disease within less than a decade, it is not very surprising that the asymptomatic (hence therapy-naïve) individuals under and over 25 years of age do not significantly differ in time of infection. In the light of this argument it appears that the age-restriction to under-25 years old is not very effective in terms of selecting recent infections. (The same argument would also apply for the intention to minimize chances of being exposed to ARVs.) Moreover, given that the over-25 years old – at least in our study - significantly differ in HIVDR frequency compared to young patients, this age-restriction may turn out to be even counterproductive in an attempt to determine representative HIVDR prevalence rates.

Considering the high frequency of HIVDR in our study population, we recommend the implementation of ART baseline resistance testing in the Mwanza area, as HIVDR prevalence in this area may have reached a critical level. In this regard, we encourage the development of cheap, robust and easy-to-use methods for detection of the commonest resistance-associated mutations as well as the regular monitoring of viral load during ART also in resource-limited settings. This would allow the selection of suitable ARVs from the available repertoire and prevent early therapy failure.

In conclusion, the results of this study demonstrate that the frequency of HIVDR in our study population is much higher than anticipated from the so-far published data of low tHIVDR levels in Tanzania and sub-Saharan Africa in general, and – even more important – that a restriction to patients <25 years of age as implemented in the current criteria of the WHO-initiated HIVDR threshold survey would have dramatically failed to detect this. The reason for this discrepancy is that the frequency of HIVDR correlated with age, for it was totally absent in young individuals, but surprisingly high in individuals being older than 25 years. Sporadic and undisclosed misuse of ARVs may have contributed to the observed high resistance rate. This indicates that the exclusive focus on transmission of HIVDR in under 25-years old as recommended by the current WHO HIVDR threshold survey, instead of a broader approach that is open to HIVDR accumulation by different mechanisms and across all age groups, may fail to adequately monitor the effects of ART role out programs on the spread of HIV drug resistance into the therapy-naive population. We therefore recommend to perform similar studies in other resource-limited areas to test whether the so far reported optimistic picture of low HIVDR prevalence in young individuals is really representative for the rest of the ART-naïve HIV-infected population.
